# Substance use assessment: comparing self-reports with objective data in a research setting

**DOI:** 10.3389/fpubh.2025.1628519

**Published:** 2026-01-12

**Authors:** Alicja Anna Binkowska, Piotr Pałczyński, Natalia Jakubowska, Jakub Czarny, Michał Raczkowski, Aneta Brzezicka

**Affiliations:** 1 Institute of Psychology, Humanitas University, Sosnowiec, Poland; 2 Neurocognitive Research Center, Institute of Psychology, SWPS University, Warsaw, Poland; 3 Institute of Forensic Genetics, Bydgoszcz, Poland

**Keywords:** drug use, self-report data, hair sample, biological data, objective data

## Abstract

Accurate assessment of substance use is essential in public health, clinical, and research settings. While self-reports are widely used, they are prone to biases such as social desirability and recall errors. Objective biological measures, such as hair toxicology, offer a longer detection window and may improve data validity. This study examined the concordance between self-reported substance use and hair toxicology results, with a particular focus on cannabis. It also explored the prevalence and predictors of underreporting, and the relationship between cannabis use patterns and THC detection in hair. Data were collected from 75 adult participants. Self-reported substance use was assessed via questionnaire, and hair samples were analyzed using liquid chromatography–mass spectrometry (LC-MS/MS) to detect drug use over the prior 3 months. Underreporting was defined as a negative self-report with a positive hair test. Results showed that 21.3% of participants underreported use of at least one substance. While group differences were not statistically significant, moderate-to-large effect sizes were observed, these effect sizes are descriptive in nature and may reflect possible discordance for substances such as MDMA and cocaine. No sociodemographic factors or alcohol use patterns significantly predicted underreporting. However, self-reported cannabis use frequency and quantity were significant predictors of THC detection, while years of use and time since last use were not. These findings highlight the limitations of relying solely on self-reports and emphasize the value of integrating objective biological measures. A combined approach improves the accuracy of substance use assessment and helps address underreporting biases, particularly in settings where legal or social pressures may influence disclosure.

## 1 Introduction

Accurate assessment of substance use is crucial for public health interventions, clinical treatment, legal proceedings, and scientific research. High-quality data on substance use is essential for understanding its effects on various aspects of human health, including brain function and cognitive processes. In research contexts, precise measurements of substance use can clarify how different substances impact brain and cognitive functions, ultimately guiding the development of effective treatment and intervention strategies.

Traditional methods of assessing substance use often rely on self-reports, where individuals disclose their substance use behaviors through questionnaires or interviews. These methods are popular due to their simplicity and cost-effectiveness. However, they are subjective and vulnerable to biases such as recall errors, social desirability, and both intentional and unintentional underreporting (1–4). These biases can compromise data accuracy, impacting research outcomes.

Intentional underreporting of drug use may occur because of the sensitive nature of the topic—participants may fear judgment, social stigma, or legal consequences from disclosing their drug use (5). Concerns about personal relationships or professional consequences can also increase the likelihood of underreporting (5). Not all underreporting is deliberate, however. Individuals might forget their drug use, misunderstand survey questions, or be unaware of exposure to contaminated or mixed drugs (5). For example, psychoactive drugs such as heroin and ecstasy (MDMA) are often mixed with other substances, including methamphetamine or new psychoactive substances (NPS) (6–8). A growing concern is the presence of fentanyl and its analogs in drugs like heroin and cocaine, which increases the risk of overdose for users unaware of the contaminants (9, 10).

Advancements in analytical techniques have introduced objective measures of substance use, such as biological sample analysis. These methods offer a more reliable assessment by detecting the presence of substances and their metabolites in bodily fluids or tissues. Common biological testing methods—such as blood, saliva, and urine tests—are well-suited for capturing recent use within a few days, making them effective for evaluating acute and recent drug effects. However, they may not be ideal for identifying long-term drug use or its residual effects (11).

Hair sample analysis provides a unique advantage in substance use research due to its extended detection window—enabling retrospective identification of drug use over several months (12–14). This feature is particularly valuable for studies that aim to assess longer-term patterns of drug use and compare them with self-reported behavior. Unlike urine, saliva, or blood samples—which primarily detect recent consumption over hours to a few days—hair offers a cumulative record of substance exposure over time, making it a more suitable choice for studies examining broader usage trends.

From a practical standpoint, hair collection is non-invasive, does not require specialized medical personnel, and is relatively easy to transport and store. Compared to saliva or urine (if not immediately tested), which can be susceptible to adulteration and often demand immediate processing or refrigeration, hair provides a stable matrix for toxicological analysis (15). Despite these advantages, hair analysis does present challenges: it is more costly than other methods, and participants may be hesitant to provide samples due to cosmetic concerns or discomfort with the amount of hair required. Previous studies have reported hair sample refusal rates ranging from 20% to over 85% in non-clinical samples in research settings (15–18).

Additionally, individual differences in hair growth and potential external contamination are known limitations, although these can be mitigated with standardized washing procedures and validated analytical protocols (19). Moreover, previous research indicates that hair sample analysis is characterized by high specificity but relatively lower sensitivity, which depends on the type of substance and the individual's history of use (20, 21, 45). High specificity ensures a low probability of obtaining false-positive results, making it relatively reliable for verifying self-reported abstinence from substance use. However, the lower sensitivity increases the risk that substances that have actually been consumed remain undetected. Hair analysis demonstrates the highest sensitivity for substances such as opioids (e.g., heroin) and the lowest for cannabis (15). Sensitivity is also influenced by the amount and frequency of substance use; it tends to be lower in cases of low-dose or infrequent consumption (22). Additionally, drug concentration in hair is affected by hair structure and melanin content (23) and may be altered by cosmetic treatments such as perming, straightening, or dyeing, which can result in gender and ethnic differences in measurement accuracy (24). It is important to note that hair toxicology cannot reliably detect very recent substance use because drugs require time to be incorporated into the growing hair shaft. As a result, consumption occurring within approximately the past week is typically not observable in hair samples, despite the method's broad retrospective detection window (22, 25).

Alternative biological matrices were considered but found less appropriate for our research objectives. Saliva testing, though highly acceptable and minimally invasive, detects drug use only within a narrow window (typically up to 48 h post-consumption) (26), limiting its utility for evaluating sustained patterns of use. Blood testing offers precise quantification but is invasive and impractical outside clinical environments. Urine testing has a moderately longer detection period and is more commonly used, yet it remains vulnerable to manipulation and is still limited in capturing chronic or historical use (11).

Given our study's aim—to examine the agreement between self-reported substance use and objective biomarkers over an extended timeframe—hair analysis provided the most appropriate balance of temporal sensitivity, data stability, and feasibility in a non-clinical research context. While we acknowledge its limitations, the capacity to detect multiple substances retrospectively over months outweighed concerns about participant burden or sample variability.

Liquid chromatography-mass spectrometry (LC-MS) is considered the gold standard for detecting drugs, including delta-9-tetrahydrocannabinol (THC) (27). Research suggests that the sensitivity of THC detection in hair depends on usage patterns: for heavy cannabis users, the sensitivity is around 80%, but it drops to 55% when distinguishing any cannabis users from non-users (28). This suggests that hair analysis alone may not accurately detect cannabinoid exposure. As Kroon et al. (29) emphasized, further research is needed to compare hair-derived samples with self-reports to better assess the validity of hair analysis for this purpose.

Despite the increasing use of hair testing in clinical and forensic contexts, relatively few studies have directly compared self-reported substance use to hair toxicology in non-treatment-seeking, research-based populations. Moreover, there remains a lack of clarity regarding which patterns of self-reported cannabis use—such as frequency, quantity, or recency—are most predictive of a positive THC result in hair. This study addresses these gaps by examining the degree of agreement between self-reported substance use and hair toxicology outcomes for four commonly used drugs: cannabis, MDMA, amphetamine, and cocaine. We focus particularly on cannabis use, testing whether THC detection in hair is predicted by frequency of use, grams consumed per week, and time since last use. In addition, we examine the prevalence of underreporting—defined as a mismatch between negative self-report and positive hair test—and assess whether individual characteristics such as sex, age, education, mental health diagnosis, and alcohol use predict this discrepancy.

By exploring these associations in a Central European sample, we aim to contribute to improving the validity of substance use assessments in research settings. This is especially important given the growing reliance on both self-report and biological measures in psychiatric and neuroscientific studies. This research is particularly significant in light of the meta-analyses by Bharat et al. (20), which, while demonstrating high overall agreement between biological and self-reported methods, highlighted important limitations—mainly, a highly heterogeneous evidence base and a sample predominantly (91%) from North America. Given that substance use patterns differ significantly between Europe and the United States (as well as across Europe itself) due to varying cultural, legal, and social influences [e.g., (30–33)], our data from Poland provide an additional perspective. By situating our findings within this broader international context, this study contributes novel insights into the accuracy of self-reporting in a distinct cultural and regulatory setting.

## 2 Materials and methods

### 2.1 Participants

Seventy-five adults completed the included assessments as part of a larger EEG project (34, 35). A total of 87 participants took part in the original study, of whom 75 agreed to provide a hair sample, resulting in a hair collection participation rate of approximately 86%. All participants from the original study completed questionnaires about substance use; however, only those who provided both hair samples and completed the self-report assessments were included in the current analyses. The study used a non-clinical, community-based sample recruited via public advertisements and social media. Participants were recruited in Poland through social media platforms (Facebook, Instagram) and via physical flyers posted at the university. Individuals who expressed interest completed an online screening form and were contacted by researchers to schedule their participation if they met the basic eligibility criteria.

The study was conducted in a laboratory at SWPS University in Warsaw, Poland. The research protocol was approved by the SWPS University Research Ethics Committee (approval no. 9/2018), and all participants gave written informed consent. Participants received personalized feedback on their IQ test performance and a sample recording of their brain's electrical activity as compensation for their participation.

Participants were recruited into two groups: regular cannabis users and a control group. Cannabis users were required to report using cannabis at least once a month for a minimum duration of 2 years (indicating regular and long-term use) and to score 12 or lower on the Cannabis Use Disorder Identification Test–Revised [CUDIT-R; (36)], completed via an online recruitment questionnaire prior to the study, indicating no signs of cannabis use disorder. The control group consisted of individuals who self-reported not using cannabis or any other drugs.

General inclusion criteria for all participants were: 21–42 years of age, Polish as a first language, and normal or corrected-to-normal vision (which was related to the behavioral computer procedure measuring cognitive functioning).

Exclusion criteria included a self-reported history of brain injury, diagnosed neurological disorders, and insufficient hair length (less than 3 cm at the posterior vertex), which was required for hair toxicology analyses. Participants also reported recent hair treatments (e.g., bleaching, dyeing, chemical straightening), this information was provided to the toxicology laboratory.

Although participants were initially recruited as self-reported cannabis users and drug-naïve controls, we analyzed the combined sample to capture a broader spectrum of substance use behaviors. This approach enabled us to assess agreement between self-reports and toxicology results across varying levels of use, offering a more ecologically valid representation of substance use patterns within a non-clinical population.

The sample (mean age 29.52 ± 5.19) consisted of 42 men (56%) and 33 women (44%). The average number of years of education among the study participants was 16.69 ± 2.06. Eleven participants (14.9%) reported having a diagnosed mental disorder. Use of sleep, sedative, or psychotropic medications was reported by 4 participants (5.3%). Average alcohol consumption [1 unit = one large beer = one glass of wine = one small glass of vodka] in the sample was as follows: less than one unit per week: 30 participants (40%); one to three units per week: 8 participants (10.7%); one unit per day: 1 participant (1.3%); 36 participants (48%) reported alcohol abstinence. Regular tobacco smoking was reported by 8 participants (10.7%), occasional smoking by 24 participants (32%), and 43 participants (57.3%) were nonsmokers. Mean cannabis duration of use was 8.6 years (SD = 5.87, MIN = 2, MAX = 31).

To contextualize external validity, the sample's educational attainment was compared to national statistics. The average number of years of education in the sample was 16.7 (SD = 2.06), which is higher than the national average for Polish adults aged 25–44. In 2023, 34.1% of Polish adults aged 25–64 had attained tertiary education, while over 93% had completed at least upper secondary education (37). Our sample was therefore skewed toward higher educational attainment. Importantly, the sample likely underrepresents individuals from rural or socioeconomically disadvantaged backgrounds. Most participants were recruited in urban Warsaw, while approximately 60% of Poland's population resides in urban areas (38).

More detailed data on participants may be found in Supplementary Table S1.

### 2.2 Self-reported substance use

Substance use was assessed with a self-reported drug history questionnaire. Participants reported the age at which they first used cannabis, years of use, frequency of use during the past 3 months, typical weekly quantity (in grams), and the time since last use. They also listed all psychoactive substances used within the past 3 months by name, allowing for open-ended reporting.

Cannabis frequency and quantity categories were adapted from prior frameworks (39, 40) but adjusted to reflect the Polish context, where cannabis is typically purchased in gram-denominated amounts on informal markets. Participants indicated their cannabis use frequency by selecting from categories of never, less than twice a year, 2–3 times per month, 1–3 times per week, 3–6 times per week, or daily use. They also reported their typical weekly quantity using gram-based categories, including less than 1 gram, 1–2 grams, 3–5 grams, and more than 5 grams per week. To strengthen the accuracy of quantity estimates, participants were shown a standardized image illustrating approximately one gram of cannabis [as used in (40)], which served as a visual reference when estimating weekly consumption. Participants also reported the time since their last cannabis use by selecting from standardized categories: less than 12 h, 12–24 h, 1–3 days, 3–7 days, 7–14 days, or more than 14 days prior to the study visit.

Alcohol use was collected as the number of standard drinks per week (1 drink = 1 large beer, 1 glass of wine, or 1 small serving of vodka) and categorized as: 0 drinks, < 1 drink, 1–3 drinks, 4–6 drinks, and 7–14 drinks per week. Tobacco use was categorized as: no use, occasional use (less than daily), or daily use. Use of other psychoactive substances (including illicit drugs and psychotropic medication) within the past 3 months was recorded through an open-ended item asking participants to list each substance consumed.

The questionnaires were administered in a quiet, private room at the research laboratory, during the same session as EEG and hair sample collection. Participants received the questionnaires in sealed envelopes and were given privacy to complete them. Once finished, they sealed the envelope before returning it. This procedure was designed to protect participants' confidentiality and increase honesty when reporting sensitive information.

### 2.3 Hair toxicological analyses

Moreover, illicit substance use over the last 3 months was examined by 3 cm-hair samples. The average concentration of each hair segment was calculated and used for the final analyses.

Hair samples were taken from the posterior vertex region of the scalp, which offers a consistent growth rate and a lower risk of external contamination. Approximately 100 mg of hair was collected (about the diameter of a standard pencil; ~6–8 mm). Hair collection occurred during the same lab session as the self-report assessments and EEG recordings. Samples were labeled using anonymous participant codes, stored in envelopes, and kept at room temperature until delivery to the Institute of Forensic Genetics in Bydgoszcz for analysis. All participants who provided hair samples supplied sufficient material for analysis; no attrition occurred due to insufficient sample volume.

Hair samples were analyzed for 512 drugs and their metabolites by an extremely sensitive and specific analytical technique—Liquid Chromatography Mass Spectrometry (LC-MS/MS).

### 2.4 Underreporting

Underreporting was defined as a case where a participant did not report the use of a given substance, but the hair toxicology analysis was positive. These cases were coded as 1, and all other cases as 0.

### 2.5 Statistical analyses

Data analysis was conducted using IBM SPSS Statistics 29.0 software. Descriptive statistics (e.g., mean and standard deviation, percentage) were calculated for demographic and substance use variables. To compare two dependent groups in terms of differences in self-reported and objective measures of psychoactive substance use, the non-parametric McNemar test was conducted. Given the relatively small sample size, the Exact McNemar Test was also applied to obtain a more precise *p*-value, overcoming the limitations of the asymptotic chi-squared distribution.

**Figure 1:**
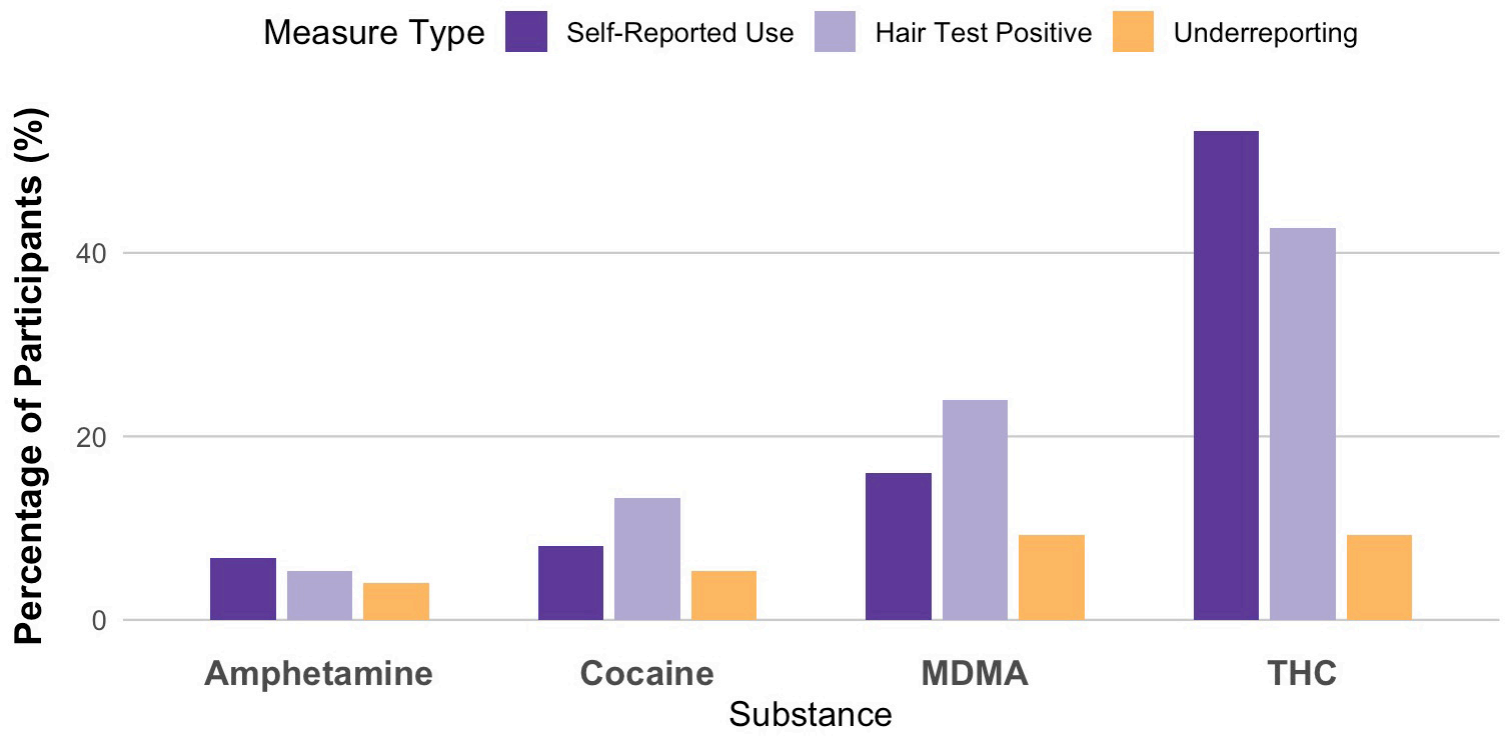
Comparison of substance use based on hair analyses, self-report, and underreporting rates for the previous 3 months (*n* = 75).

Additionally, the χ^2^ statistic was calculated to evaluate the overall agreement between the methods, accounting for both asymmetrical and symmetrical differences—symmetrical changes being those that might not be detected by the McNemar test alone. Effect size coefficients were also computed to provide insight into the difference between changes in both directions, relative to their total occurrence, offering a more nuanced understanding of the nature of the discrepancies. The Phi(Φ) effect size (Φ, capturing the strength of the association between self-reports and hair test outcomes), and *g*-Cohen coefficients (highlighting the magnitude and direction of discrepancies between changes in both directions relative to their total occurrence). For phi effect sizes: small effect size; Φ = 0.3: moderate effect size; Φ = 0.5: large effect size. For the Cohen's *g* coefficient values of approximately g = 0.05–0.1 are considered negligible, g = 0.1–0.3 small, g = 0.3–0.5 moderate, and values above g = 0.5 represent a large effect. When *p*-values are non-significant, effect sizes are treated as descriptive indices only. The statistical significance threshold was set at α ≤ 0.05, and the statistical trend threshold at α ≤ 0.1. Unless otherwise specified, percentages reported in the text, Table 1, and Figure 1 are calculated relative to the total sample (*N* = 75).

**Table 1:** Comparison between self-reports and hair toxicology results for substance use in the past 3 months (*n* = 75).

**Substance**	**Positive self-report *n* (%)**	**Positive hair test *n* (%)**	**Underreporting n (%)**	**χ^2^ (McNemar)**	***p* (exact McNemar)**	**Effect size Φ [95% CI]**	**Effect size *g*-Cohen [95% CI]**
THC	40 (53.3)	32 (42.7)	7 (9.3)	13.783	0.134	0.429 [0.215, 0.614]	0.364 [-0.053, 0.76]
MDMA	12 (16)	18 (24)	7 (9.3)	35.861	0.07	0.692 [0.478, 0.889]	−0.75 [−1, −0.2]
Amphetamine	5 (6.7)	4 (5.3)	3 (4)	2.282	1	0.175 [0.024, 0.619]	0.143 [−0.667, 1]
Cocaine	6 (8)	10 (13.3)	4 (5.3)	42.391	0.125	0.752 [0.492, 0.936]	−1 [−1, −1]
LSD	2 (2.7)	1 (1.3)		36.993	1	0.702	
Antiseizure	1 (1.3)	1 (1.3)		0.014	1	0.014	
SSRI	4 (5.3)	2 (2.7)		36.473	0.500	0.697	

Effect size thresholds: ϕ = 0.10 (small), 0.30 (moderate), 0.50 (large); Cohen's g = 0.10 (small), 0.30 (moderate), 0.50 (large).

## 3 Results

### 3.1 Self-reported substance use vs. hair toxicological analyses

In general, 21.3% (*N* = 16) of participants underreported their use of at least one drug. A total of four substances were analyzed: THC, MDMA, amphetamine, and cocaine. We did not analyze or interpret data on LSD, SSRIs, or antiseizure medication due to minimal occurrence. The results are presented in terms of positive self-reports, positive hair toxicology, and underreporting (i.e., cases where hair tests were positive but self-reports were negative).

For THC, underreporting was observed in 9.3% of participants of total sample. While 53.3% of the sample self-reported cannabis use, only 42.7% showed a positive result in hair testing. This discrepancy may reflect the known limitations in the sensitivity of THC detection in hair, particularly for occasional users. Although the McNemar test did not yield statistically significant results (χ^2^ = 13.783, *p* = 0.134), the moderate effect size indicated a moderate discrepancy between the methods [Φ = 0.429, 95% CI [0.215, 0.614]; Cohen's g = 0.364, 95% CI [−0.053, 0.76]]. However, because the *p*-value is non-significant, this discrepancy is treated as descriptive.

In the case of MDMA, 9.3% of participants also underreported use. Self-reported use was lower (16%) compared to positive hair test results (24%). The McNemar test approached statistical significance (χ^2^ = 35.861, *p* = 0.070), and the effect size was large [Φ = 0.691, 95% CI [0.478, 0.889]], with Cohen's g indicating a large discrepancy [Cohen's g = −0.75, 95% CI [−1, −0.2]]. Although not statistically significant, the pattern points to a trend of underreporting for this substance that may be of practical relevance.

Amphetamine use was underreported by 4% of participants. Here, positive self-reports (6.7%) and positive hair tests (5.3%) were more closely aligned. The McNemar test was non-significant (χ^2^ = 2.282, *p* = 1.000), and effect sizes were small [Φ = 0.174, 95% CI [0.024, 0.619]; Cohen's g = 0.143, 95% CI [−0.667, 1]], indicating a high degree of consistency between subjective and objective data for this substance.

For cocaine, 5.3% of participants failed to self-report use that was detected via toxicology. Hair test results indicated a higher prevalence (13.3%) compared to self-reports (8%). Despite the small number of positive cases, the discrepancy yielded effect size [Φ = 0.752, 95% CI [0.492, 0.936]] and Cohen's g value [Cohen's g = −1, 95% CI [−1, −1]], though the McNemar test did not indicate statistical significance (χ^2^ = 42.391, *p* = 0.125). It is important to note, that the confidence interval for Cohen's g was calculated as [−1, −1], likely reflecting complete directional asymmetry and the small number of discordant cases (*N* = 4). This result should be interpreted with caution, as the confidence interval does not capture uncertainty under such conditions. Overall, these values suggest a substantial imbalance between self-reported and toxicological data for cocaine, consistent with potential underreporting, even in the absence of statistical significance. This pattern should, however, be interpreted as descriptively informative, given the small number of discordant cases. These findings are summarized in Table 1 and visualized in Figure 1, with percentages reported relative to the total sample (*N* = 75). The figure presents aggregated data across all participants for each substance. Bars for self-reported use and positive hair test results reflect the overall prevalence in the sample (i.e., what percentage of participants either reported use or tested positive). In contrast, the underreporting bar shows the proportion of participants who tested positive but did not report use. The full numerical cross-tabulations are available in Supplementary Table S2, with percentages calculated within each self-report group (i.e., relative to the total number of participants who reported “Yes” or “No” for each substance).

### 3.2 Potential predictors of underreporting

To assess whether selected individual characteristics predicted underreporting, a series of binary logistic regression analyses was conducted. Analyses were conducted for the following predictors: sex, age, diagnosed mental disorder, years of education, and alcohol consumption.

The analysis revealed that the omnibus tests of model coefficients for all predictor variables for the variable underreporting, namely: sex (χ^2^ = 1.956; *p* = 0.162), age (χ^2^ = 0.486; *p* = 0.486), diagnosed mental disorders (χ^2^ = 1.200; *p* = 0.273), years of education (χ^2^ = 0.838; *p* = 0.360), and alcohol consumption (χ^2^ = 1.615; *p* = 0.204), yielded non-significant results. The chi-square values of the models, along with their significance levels, are presented in parentheses above.

### 3.3 Relationship between self-reported cannabis use patterns and THC detection

To explore whether self-reported cannabis use patterns predicted THC detection in hair, a series of binary logistic regression analyses were conducted, with hair test results (positive vs. negative) serving as the dependent variable.

The explanatory variables were: self-reported frequency of cannabis use (MJ Use Freq; χ^2^ = 25.448; *df* = 1; *p* < 0.001); reported number of grams of cannabis used per week (MJ g/week; χ^2^ = 9.038; *df* = 1; *p* = 0.003); and reported last use of cannabis (Last MJ Use; χ^2^ = 0.259; *df* = 1; *p* = 0.071). The collective test of model coefficients for the last of these explanatory variables showed results at a level indicating a statistical trend.

Self-reported frequency of cannabis use was a significant predictor of a positive THC result in hair [*Wald* = 14.658; *df* = 1; *p* < 0.001; Exp(β) = 1.592], meaning that a one-unit increase in frequency of cannabis use (MJ Use Freq) increased the likelihood of a positive THC result in hair by a factor of 1.592; this relationship explains approximately 38% of the variance (Nagelkerke *R*^2^ = 0.386).

Similarly, the reported number of grams of cannabis consumed per week (*MJ g/week*) was also a significant predictor [*Wald* = 7.509; *df* = 1; *p* = 0.006; Exp(β) = 2.736], with each additional gram increasing the likelihood of THC detection by a factor of 2.736. This model explained approximately 21% of the variance (Nagelkerke *R*^2^ = 0.215).

Two additional predictors—duration of cannabis use (χ^2^ = 0.163, *df* = 1, *p* = 0.686) and time since last cannabis use (χ^2^ = 2.982, *df* = 1, *p* = 0.084)—did not yield statistically significant results. Results are summarized in Table 2.

**Table 2:** Logistic regression analysis of self-reported cannabis use patterns as predictors of THC detection in hair.

**Variables**	**B**	**SE**	**Wald**	**df**	**p**	**Exp(B)**	**Nagelkerke *R*^2^**
Intercept	−3.259	0.911	12.806	1	< 0.001	0.038	
MJ Use Freq	0.465	0.122	14.658	1	< 0.001	1.592	0.386
Intercept	−1.607	0.760	4.470	1	0.034	0.200	
MJ g/week	1.007	0.367	7.509	1	0.006	2.736	0.215

Intercept, baseline result from hair test indicating the presence of psychoactive substances; MJ Use Freq, frequency of cannabis use (never; less than twice a year; 2–3 times per month; 1–3 times per week; 3–6 times per week; daily); MJ g/week, grams of cannabis used per week (< 1 g; 1–2 g; 3–5 g; >5 g per week).

## 4 Discussion

The present study provides important insights into the relationship between self-reported substance use and objective biological measures, such as hair toxicology analysis, particularly for cannabis use. These findings build upon the work of Bharat et al. (20), who conducted a meta-analysis examining the overall agreement between self-reports and biological measures.

Bharat et al. (20) found a generally high level of agreement, though it's crucial to recognize that the evidence base in their study was largely based on North American participants, a region with unique cultural and legal contexts for substance use. In contrast, our study from Poland found descriptivediscrepancies between self-reported substance use and hair toxicology results, with effect sizes suggesting possible patterns for certain substances, such as MDMA and cocaine, where underreporting could be more prevalent due to social stigma or legal concerns.

The finding that approximately 21.3% of participants underreported their drug use, as determined by discrepancies between self-reports and hair toxicology, suggests a substantial level of underreporting and aligns with existing literature on the limitations of self-reported data in substance use research (22, 41). This underreporting is a critical issue in research and can affect data quality, indicating that while not a majority, a notable portion of participants did not fully disclose their substance use. Such discrepancies highlight the potential limitations of relying exclusively on self-report measures.

It is important to note that the small sample sizes across several substance categories limit the study's statistical power to detect significant differences, potentially contributing to the non-significant *p*-values. To account for this, we reported effect sizes (Φ and Cohen's g) along with 95% confidence intervals for each substance. This approach allows the data to be interpreted in ways that extend beyond simple significance testing and provides descriptive insight into potential patterns of interest. Despite the lack of statistical significance in many χ^2^ comparisons, effect sizes offer valuable insight into the strength of observed discrepancies and are used here in a descriptive rather than inferential manner. For MDMA, we observed a trend toward significance and a large effect size, suggesting differences between self-reports and objective measures. For cocaine, a similarly large effect size was found despite non-significant *p*-values, which may be consistent with possible underreporting biases, although these findings should be interpreted with caution given the small number of positive and discordant cases. These trends may reflect the complexity of self-reporting, particularly for substances that carry social or legal stigma (42, 43). In the case of amphetamine, the effect size was small, indicating a potential alignment between self-reports and hair samples—though the small sample size should again be noted as a limitation.

This rate of underreporting is consistent with previous studies that have identified both intentional and unintentional biases in self-reporting, driven by various psychological, social, and contextual factors (44). Several factors likely contributed to the observed underreporting in our study. One likely contributing factor is social desirability bias, where participants may downplay their substance use to present themselves in a more favorable light. In research settings—especially those involving sensitive topics—participants may fear judgment or stigmatization, leading to underreporting (5). Another plausible factor is the fear of legal or social consequences. Even in confidential studies, participants may hesitate to disclose illegal behaviors. This concern may be particularly relevant in countries like Poland, where drug use is both legally restricted and socially stigmatized.

Unintentional underreporting may additionally arise from recall difficulties, especially when substance use is sporadic, irregular, or occurred in social contexts involving intoxication (e.g., parties). Participants may struggle to remember frequency, quantity, or timing accurately. Additionally, involuntary exposure to adulterated drugs may explain some cases of underreporting. For instance, MDMA and other party drugs are often mixed with methamphetamine or other psychoactive compounds (6, 7), meaning users might test positive for substances they did not knowingly consume.

While the effect sizes suggest possible differences between self-reports and objective measures, our statistical analyses of potential predictors of underreporting did not reveal any significant associations with sociodemographic variables or alcohol consumption. Neither sex, age, education level, mental health status, nor drinking behavior significantly predicted underreporting, but rather influenced by personal, social, or contextual factors. It may also point to recall issues, particularly in cases of infrequent or incidental drug use.

In the case of THC, underreporting was observed in 9.3% of participants. Interestingly, a higher percentage of participants self-reported cannabis use (53.3%) than those who tested positive via hair analysis (42.7%), which may point to limitations in the sensitivity of hair toxicology in detecting cannabis use. Previous studies have shown that THC detection in hair varies considerably depending on use intensity—while sensitivity can reach around 80% for heavy users, it drops to approximately 55% for occasional users (28). The presented findings are consistent with previous research indicating that hair toxicology demonstrates high specificity but significantly lower sensitivity, particularly among occasional users (20–22). Therefore, negative hair test results may reflect methodological limitations of hair analysis, particularly among infrequent users. At the same time, the presence of underreporting in a subset of participants underscores the need to incorporate objective measures alongside self-reports. These findings suggest that neither method may be sufficient on its own—while hair testing may miss some true cases of use, self-reports are susceptible to bias. Taken together, this pattern highlights the importance of a multi-method approach to improve the accuracy and completeness of cannabis use assessment.

The study also examined self-reported cannabis use patterns as predictors of THC detection in hair. Frequency of use and quantity consumed (grams per week) were significant predictors, whereas duration of use and time since last use were not. These results are consistent with prior research indicating that recent and cumulative exposure are more relevant for hair detection than lifetime use history.

It should be noted that 12 participants (13.8%) from the larger EEG project (the original study from which the data were drawn) declined to provide hair samples. Despite assurances of confidentiality and the non-invasive nature of the procedure, the primary reason for refusal was cosmetic, particularly a reluctance to cut visible hair. While this attrition reduced the final sample size for hair-based measures, the refusal rate in our study is relatively low when compared to prior research. Previous studies have reported hair sample refusal rates ranging from 20% to over 85% in community-based or non-clinical populations in research settings (15–18). Nevertheless, future research should consider incorporating alternative biological sampling methods (e.g., saliva or urine), which may enhance participation rates without compromising data quality.

In light of these results, it is essential for future research to address the challenges of underreporting and identify the factors contributing to discrepancies between self-reports and biological measures. For example, future studies could investigate the impact of survey format (e.g., anonymous online questionnaires vs. face-to-face interviews) or perceived confidentiality on response accuracy. Expanding the sample to include participants from more diverse cultural and geographic contexts would also help to clarify whether certain populations are more prone to underreporting.

These findings underscore the importance of integrating objective measures such as hair toxicology to supplement self-reported data—especially for substances that carry a higher risk of underreporting. At the same time, self-reports offer valuable contextual information, such as usage motives or settings, that biological measures cannot capture. Therefore, a combined approach may be the most informative. It is important to acknowledge, however, that biological testing methods—particularly hair analysis—require specialized equipment and can be costly, which may limit their use in large-scale research.

In conclusion, this study highlights the complexities of substance use assessment and the importance of triangulating methods to improve accuracy. Despite limitations such as small sample size and the exclusive use of hair testing, the results contribute to ongoing efforts to improve the reliability of substance use data—especially in underrepresented populations and culturally specific contexts.

## Data Availability

The data that support the findings of this study are available upon request from the corresponding author. Requests to access these datasets should be directed to Alicja Anna Binkowska, alicja.binkowska@humanitas.edu.pl.
