# Supplementary material for: Substance use assessment: comparing self-reports with objective data in a research setting
Source: Front Public Health. 2026 Jan 12;13:1628519. doi: 10.3389/fpubh.2025.1628519 (PMC12832719; doi:10.3389/fpubh.2025.1628519)
Supplement: Supplementary file 1 [file Data_Sheet_1.pdf]

Supplementary material

Table 1S. Characteristics of the study group

Category	Category division	Quantity	Quantity in %
**Sex**	Female	33	44%
	Male	42	56%
**Use of sleeping, calming, or psychotropic medications**	Yes	4	5.3%
	No	71	94.7%
**Diagnosed mental disorder**	Yes	11	14.9%
	No	63	85.1%
**Average alcohol consumption**	Abstinence	36	48%
	Less than one unit per week	30	40%
	1-3 units per week	8	10.7%
	1 unit daily	1	1.3%
**Frequency of tobacco smoking**	None (zero)	43	57.3%
	Occasionally	24	32%
	Regularly	8	10.7%
**Frequency of cannabis use**	None (zero)	19	25.3%
	Less than twice a year	6	8%
	2-3 times a month	2	2.7%
	1-3 times a week	15	20%
	3-6 times a week	15	20%

	Daily	18	24%
**Dose in grams per week**	Less than 1 g	18	34.6%
	1 - 2 grams	15	28.8%
	3 - 5 grams	16	30.8%
	> 5 grams	3	5.8%

Note: 1 unit = one large beer = one glass of wine = one small glass of vodka

Table 2S. Cross-tabulation of self-reported substance use and hair toxicology results (n = 75).

Substance	Self-reported use	Positive hair test n (%)	Negative hair test n (%)	Total n
**THC**	Yes	25 (62.5%)	15 (37.5%)	40
	No	7 (20.0%)	28 (80.0%)	35
**MDMA**	Yes	11 (91.7%)	1 (8.3%)	12
	No	7 (11.1%)	56 (88.9%)	63
**Amphetamine**	Yes	1 (20.0%)	4 (80.0%)	5
	No	3 (4.3%)	67 (95.7%)	70
**Cocaine**	Yes	6 (100.0%)	0 (0.0%)	6
	No	4 (5.8%)	65 (94.2%)	69
**LSD**	Yes	1 (50.0%)	1 (50.0%)	2
	No	0 (0.0%)	73 (100.0%)	73
**Antiseizure**	Yes	1 (100.0%)	0 (0.0%)	1
	No	0 (0.0%)	74 (100.0%)	74
**SSRI**	Yes	2 (50.0%)	2 (50.0%)	4

	No	0 (0.0%)	71 (100.0%)	71

Note. “Positive hair test” refers to a positive toxicology result for the given substance. “Underreporting” corresponds to cells with a negative self-report and a positive hair test result (row “No”, column “Positive hair test”).
